# Management of a Multi-Level Forearm Microsurgical Reconstruction and the Following Transfusion-Related Acute Lung Injury

**DOI:** 10.7759/cureus.10385

**Published:** 2020-09-11

**Authors:** Aydin Budeyri, Betul Kocamer Simsek, Mumtaz Murat Yardimci, Recep Anlatici, Mehmet C Cankus

**Affiliations:** 1 Department of Orthopaedics and Traumatology, Sanko University, Gaziantep, TUR; 2 The Shoulder Center, Baylor University Medical Center at Dallas, Dallas, USA; 3 Department of Anesthesiology and Reanimation, Sanko University, Gaziantep, TUR; 4 Department of Cardiovascular Surgery, Sanko University, Gaziantep, TUR; 5 Department of Plastic Surgery, Sanko University, Gaziantep, TUR

**Keywords:** microsurgery, multilevel forearm injury, multilevel forearm arterial reconstruction, saphenous vein graft arterial reconstruction, trali, blood transfusion, acute lung injury, traumatic multilevel forearm amputation, deep palmar arterial arch technique for limb salvage, microsurgical management of severe segmental arterial tissue loss

## Abstract

Transfusion-related acute lung injury (TRALI) is a clinical syndrome characterized by acute respiratory distress following blood transfusion. This case-based technical report documents a case on the management of a multi-level forearm microsurgical reconstruction and the following TRALI syndrome that developed shortly after blood transfusion in a 29-year-old male. Multilevel microsurgical revascularization was performed via saphenous vein autograft arterial reconstruction from the proximal 1/3 ulnar artery to the ulnar side of the deep superficial palmar arterial arch. TRALI was resolved with intensive care unit monitoring and treatment. There are several reports of TRALI in literature, as well as proposed mechanisms of pathogenesis, however, no case on the management of a multilevel forearm arterial reconstruction via a long saphenous vein autograft and associated TRALI syndrome have been reported.

## Introduction

Transfusion-related acute lung injury (TRALI) is a clinical complication characterized by acute respiratory distress following blood transfusion. TRALI has been associated with all types of plasma-containing blood products, including rare reports of intravenous immunoglobulin (IVIG) and cryoprecipitate. The incidence of TRALI has been estimated to be 1 in 1,120 cellular blood components and 1 in 5,000 components in whole blood [1]. It is difficult to define this clinical syndrome due to the variability in reporting systems worldwide [2]. This discrepancy has major implications for the timely recognition and treatment of TRALI in emergency situations. Indeed, a clinical look-back investigation into 36 patient charts revealed several reactions to transfusions from one donor that were misdiagnosed or underreported for two years [3].

TRALI is recognized as a leading cause of transfusion-related morbidity and mortality rates (approximately 9%) [2]. Majority of patients require ventilatory support, however, the lung injury is generally transient with partial oxygen (pO_2_) levels returning to pre-transfusion levels within 48-96 hours and chest X-ray (CXR) showing no major abnormalities within 96 hours. Symptoms of TRALI typically develop during or within six hours of transfusion. Patients present with the rapid onset of dyspnea and tachypnea. Other clinical symptoms include fever, cyanosis, and hypotension. Additionally, respiratory distress is revealed by clinical examinations of arterial blood gas parameters (PaO_2_/FiO_2_ <300 mmHg), and pulmonary crackles may be present with no signs of congestive heart failure or volume overload. CXRs display evidence of bilateral pulmonary edema not associated with heart failure (non-cardiogenic pulmonary edema), including bilateral patchy infiltrates, which may rapidly progress to complete "white out" that is indistinguishable from acute respiratory distress syndrome (ARDS) [1,2]. In summary, since TRALI is acute on-setting and primarily revealing pulmonary symptoms and findings contrary to the primarily multi-organ symptoms and findings of ARDS, and contrary to the latest stage revealing pulmonary symptoms and findings of ARDS, with the support of the regarding literature reference included in our manuscript, we were able to confirm a TRALI diagnosis for this unique case.

## Case presentation

**Figure 1:**
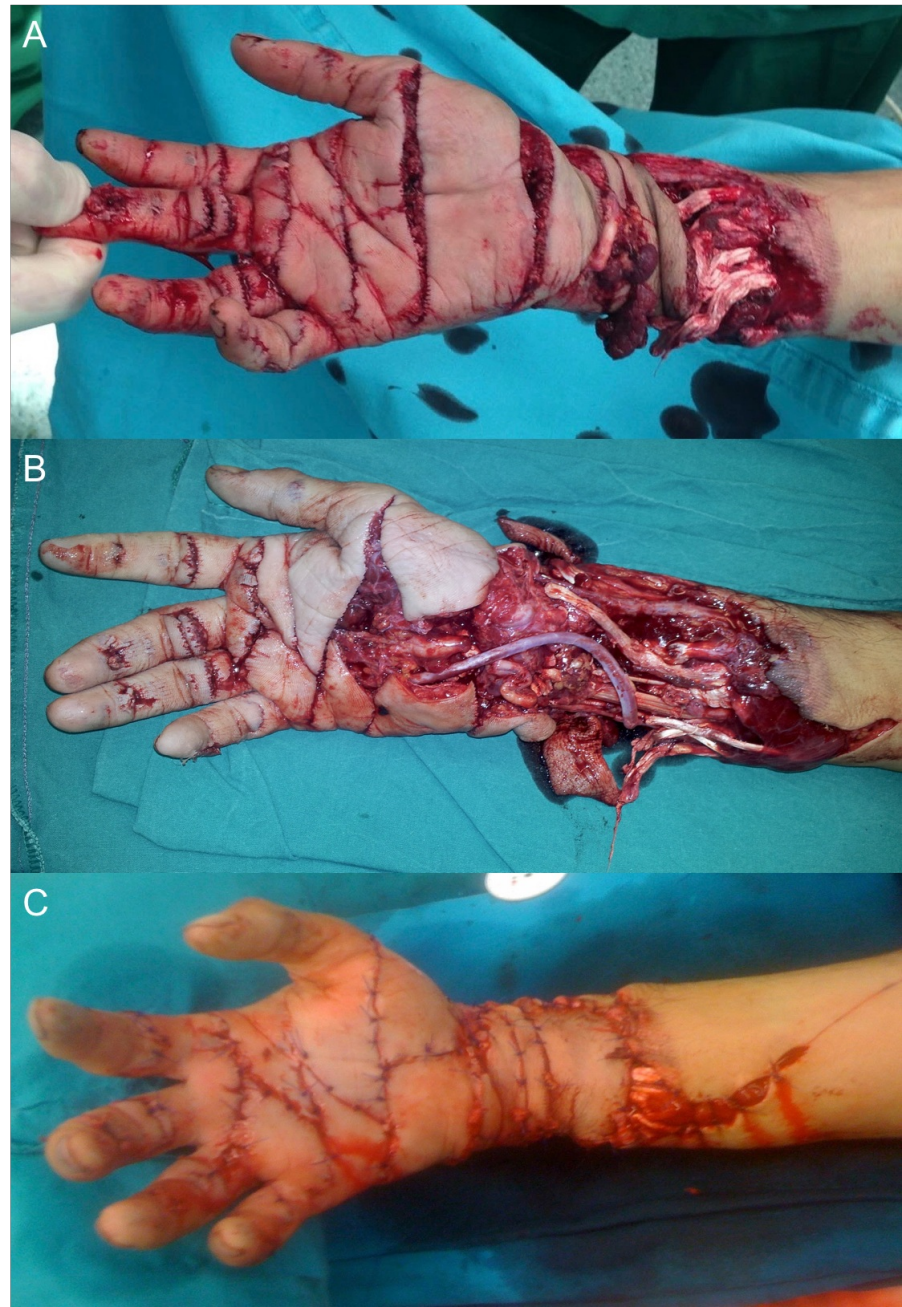
Preoperative, intraoperative and postoperative volar view of the patient's hand and forearm A.  Preoperative volar view of the multilevel amputation of the patient's right hand after sustaining injuries from a textile slicing machine revealing the loss of arterial hand perfusion and tissue turgor tonus. B. Intraoperative volar view of the patient's hand following the microsurgical saphenous vein autograft arteral defect reconstruction from ulnar artery to the ulnar side of the deep palmar arterial arch in Guyon's canal. C. Postoperative volar view of the patient's hand revealing the established arterial hand perfusion and tissue turgor tonus.

**Figure 2:**
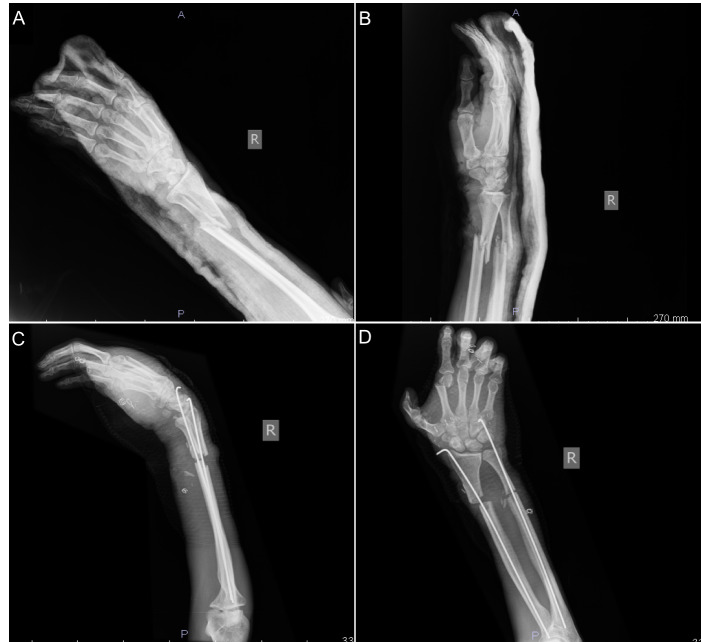
Right forearm and hand preoperative and early postoperative radiologic views A. Antero-posterior radiologic view of the patient's right hand and forearm showing the multilevel amputation injuries and open fractures. B. Lateral radiologic view of the patient's right hand and forearm showing the multilevel amputation injuries and open fractures. C, D. Postoperative radiologic views of the patient's right hand and forearm following temporary percutaneous intramedullary fixation for open fractures.

A 29-year-old male was referred to our emergency care unit at the third hour of his injury. He had experienced an avulsion amputation of his dominant right hand and forearm resulting from a textile slicing machine (Figure 1A, 2).

Along with the crush and volar skin avulsion amputation injuries and open fractures as observed in the right and hand forearm x-rays (Figure 2), he also experienced a transection of the radial and ulnar arteries and nerves, median nerve, cephalic vein, finger and wrist flexors. The patient did not have arterial bleeding in his fingers and his hand was cold, ischemic, and had lost its tonus. His vital signs were stable.

At the emergency unit, an open fracture and amputation treatment procedure began with an intensive sterile wound irrigation, debridement, dressing, and cast stabilization. He was administered an intravenous (i.v.) antibiotic prophylaxis consisting of cefazoline sodium, metronidazole, and gentamycin. Tetanus prophylaxis was also administered with tetanus vaccination since he had not been immunized within the last five years. Moreover, he received a subcutaneous administration of enoxaparin sodium and i.v. administration of dextran with pentoxifylline. Accurate analgesic medication and antiulcer prophylaxis were delivered. The patient was then taken to the orthopedic operation room for a sterile first look debridement, temporary intramedullary osteosynthesis and a microsurgical reconstruction procedure (Figure 1A).

Following operating room sterile irrigation and debridement, temporary percutaneous intramedullary osteosynthesis of the radius and ulna and flexor tendon repairs, neurovascular structures of the fingers, palm and wrist were intraoperatively re-explored under microscopic vision. Ulnar digital neurovascular bundle of the thumb, radial neurovascular bundles of the second and third finger, ulnar neurovascular bundles of the fourth and fifth finger were diagnosed to be intact. At the 11th hour post-injury, the patient had a reliable arterial circulation in all fingers with normal capillary refilling following the microsurgical end-to-end reversed saphenous vein autograft arterial reconstruction from the middle third ulnar artery to the ulnar side of the deep palmar arterial arc in the Guyon's canal. This microsurgical reconstruction and the end-to-end vascular diameter adaptation combinations successfully enabled the whole digital arterial circulation in all fingers without reconstructing every digital artery in all fingers at all sliced levels. No additional digital neurovascular repairs were performed in order to not to extend the surgery time and increase the risks of the anesthesia. Subsequently, microsurgical epineural median nerve repair under wrist flexion was performed (Figures 1B-C-D). Finally, warm saline irrigation, loose skin closure, sterile dressing, and wrist flexed forearm casting was performed. Digital nerves and arteries were microscopically explored. Radial artery was diagnosed irreparable according to the trauma mechanism, extremely severe artery defect, and unreliable proximal arterial perfusion.

**Figure 3:**
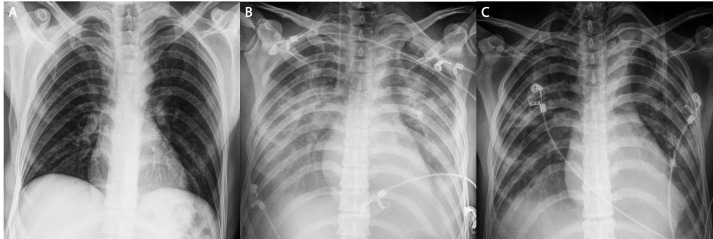
Antero-posterior chest x-rays A. Preoperative normal chest x-ray of the patient B. Postoperative chest x-ray showing extensive and diffuse bilateral pulmonary infiltrates that resulted in the diagnosis of TRALI following blood transfusions C. Postoperative chest x-ray after TRALI was resolved through a combination of adequate ventilatory support and appropriate intensive care. The chest x-ray shows a return to normal and diffuse pulmonary infiltrates are no longer detectable.

No intraoperative complications were recorded and the patient was transferred to the intensive care unit (ICU) with an operative duration of 18 hours. The preoperative anteroposterior (AP) chest radiogram of the patient displayed no irregularities (Figure 3A). 

Post the replantation surgery, the patient was monitored in the ICU for clinical and vital evaluation. The hemoglobin (Hb) level of the patient was 8.7 mg/dL in the first hour and 2 units of erythrocyte suspension (ES) along with 2 units of fresh frozen plasma (FFP) were administered. Minor bleeding from the capillaries in the wound was observed 24 hours later, with a Hb level of 8.6 mg/dL. Hence, 2 units of ES and 2 units of FFP were administered. The patient’s Hb level was 6.9 mg/dL 46 hours postoperatively and capillary bleeding had slowed down, but in order to guarantee vital support, 2 units of ES, 2 units of FFP, and 1 unit of complete blood transfusion were administered to the patient within eight hours. The patient’s respiratory irregularities occurred throughout this transfusion period. Tachypnea was detected with a measurement of 43 breathes/minute. Oxygen was continuously delivered via a mask, but the patient’s peripheral capillary oxygen saturation (spO_2_) was 62%, the pH was 7.52, carbon dioxide partial pressure (pCO_2_) was 29 mmHg, and oxygen partial pressure (pO_2_) was 31.9 mmHg in the arterial blood gas analysis. The patient was immediately intubated, and mechanical ventilation was begun with synchronized intermittent mandatory ventilation (SIMV) with pressure control and pressure support ventilation modes. The patient was monitored and evaluated closely. The mechanical ventilation parameters were adjusted as follows: (1) positive-end expiratory pressure (PEEP) was set to 5 above PEEP 20, (2) PEEP support was set to 8, and (3) the fraction of inspired oxygen was set to 50%. In the arterial blood gas analysis following the first three hours of intubation, the analysis profile was as follows: (1) pH was 7.49, (2) pCO_2_ was 32.4 mmHg, (3) pO_2_ was 109 mmHg, and (4) spO_2_ was 98%. Diffuse bilateral lung infiltration was present in the AP chest radiograms while the PaO_2_/FiO_2_ level was 218 mmHg. This respiratory profile had appeared shortly after the transfusion leading to the diagnosis of TRALI (Figure 3B).

Intravenous prednisolone was administered as a 1 mg/kg bolus and continuously at 20 mg/kg, while diuretics were avoided. AP chest radiograms and arterial blood gas analyses were screened twice daily. The decision to cease intubation was made on the fifth day of intubation when intense and diffuse infiltration observed in the AP chest radiograms was diagnosed as resolved (Figure 3C).

After the first three hours of extubation, the arterial blood gas analysis profile was as follows: (1) pH was 7.38, (2) pCO_2_ was 34.6 mmHg, and (3) pO_2_ was 84.7 mmHg. There were no further complications in the following days and the patient was transferred to the orthopedics clinic for additional wound care and routine post-replantation follow-ups.

## Discussion

TRALI has been documented in critically ill and cardiac surgery patients, who have also been identified as risk groups for this injury. However, there is a lack of literature that reports TRALI cases occurring with blood transfusions, specifically in relation to surgical procedures such as upper extremity replantation and/or reconstruction and microsurgeries. Additionally TRALI, as reported in this case and by others [4], also threatens patients who do not present with easily detectable signs. The underreporting of TRALI is likely related to the complexity of identifying TRALI and differentiating it from related complications such as transfusion-associated circulatory overload (TACO) and acute respiratory distress syndrome (ARDS). Therefore, it is imperative that the literature on this issue is expanded, especially in light of several reports observing the incidence of TRALI in relation to inadequate hemovigilance of blood products [2,5,6].

TRALI essentially involves an immune reaction to the transfusion that eventually leads to pulmonary edema, respiratory distress and failure, and in some cases death. Investigations of donor blood have revealed elevated levels of transfused donor antibodies specific for granulocytes and/or human leukocyte antigens (HLA) I and/or II as mediators of the recipient immune reaction. The development of TRALI has also been described with a two-event model including (1) the activation of endothelium in the lungs towards a proinflammatory state and (2) the interaction of primed polymorphonuclear lymphocytes (PMNs) with the activated endothelium to induce the robust TRALI response [7]. This two-event model emphasizes the role of the endothelial cells. Histopathological findings from the earlier stages of TRALI have demonstrated endothelial cell swelling and capillary congestion that progresses to filling the alveolar spaces with fluid. Furthermore, it is necessary to consider transfused immune products alongside specifics of the recipients’ physiology in light of a report from the Netherlands that reported antibodies in the majority of large-volume donor products. These antibodies were proven to be incompatible with the recipient - and leading to TRALI - in approximately half of the cases, but not all [8]. Additionally, TRALI is also thought to be caused by non-antibody-mediated factors through the accumulation of chemokines, cytokines, and proinflammatory mediators in the blood storage process [9].

Interestingly, the TRALI cases in the study from the Netherlands included blood donations from females [8] - an effect related to elevated levels of alloimmunization with a history of pregnancy [5]. Indeed, the American Red Cross surveillance data on TRALI cases from 2003 to 2005 found that the majority of cases in their study involved blood transfusion products from a female donor [6]. One clear conclusion from these findings is to use only male-donor blood or females with no history of pregnancy to minimize the risk of developing TRALI. This strategy for TRALI reduction was implemented in the United Kingdom from 1996 to 2006. In this scheme, only male donors were included for FFP and plasma for platelet (PLT) production, which resulted in a significant reduction in risk for TRALI from 36 cases in 2003 to 10 cases in 2006 [10]. In our case, the donor was a 28-year-old male, which highlights the importance of considering donor screening as well as the particulars of recipient physiology when examining protocols for TRALI susceptibility.

This case-based technical report documents a case of TRALI following blood transfusion after surgical reconstruction of a completely amputated right forearm and hand in an adult male patient. The patient was administered 10 units of blood components intraoperatively and underwent one blood transfusion over 46 hours postoperatively. Respiratory irregularities were observed throughout this transfusion period, based on which TRALI was diagnosed and the patient was intubated. The patient received treatment with prednisolone, and the TRALI case was determined as resolved after five days of intubation.

In a previous report of a case of upper extremity replantation, the surgical procedure was performed five hours after injury, TRALI developed after 36 units of blood products were transfused postoperatively and pulmonary complications developed in the early postoperative period [4]. Conversely, in our case, the surgical procedure was initiated three hours after injury, 10 units of blood products were administered, one blood transfusion was performed, and the pulmonary complications were also observed in the early postoperative period. 

A review of the literature on TRALI cases revealed several studies that examined the impact of donor blood, especially when the source is a female with a history of pregnancy [5,6,8,10]. This explanation for the development of TRALI in the case reported here is inconclusive as the blood donor was a young adult male. Therefore, it is necessary for future research to elucidate the possible causes that lead to the development of TRALI. Lastly, it is crucial that reporting of TRALI due to blood transfusions become a common practice since it can inform medical practitioners and researchers of this potentially fatal syndrome.

## Conclusions

TRALI is an adverse and possibly fatal reaction to blood transfusion that occurs within six hours of the transfusion. It is the leading cause of morbidity and mortality related to blood transfusions. The proposed mechanisms of pathogenesis of TRALI are yet to be fully expounded. Lastly, it is crucial that reporting of TRALI due to blood transfusions become a common practice since it can inform medical practitioners and researchers of this potentially life-threatening syndrome, which can lead to timely recognition, diagnosis and treatment in emergency situations.

Thorough microsurgical examination during the preoperative and intraoperative periods is essential to be able to create an accurate and reliable microsurgical reconstruction strategy. Microsurgical saphenous vein autografting for the severe arterial defects of the forearm and for the multilevel amputation of the hand is an effective technique. Utilization of the ulnar side of the deep palmar arterial arc for the microsurgical reanastomosis is an effective shortcut technique to revascularize the multislice devascularized fingers eliminating the need of reanastomosing every digital artery in all slice levels. End-to end adaptation of the saphenous vein graft's distal orifice diameter and the ulnar side of the deep palmar arterial arc's proximal orifice diameter in the Guyon's canal is a safe strategy in selected severe cases. 
